# Oxidative Stress and Apoptotic Markers in Goats Naturally Infected with *Mycobacterium avium* subsp. *paratuberculosis*

**DOI:** 10.3390/pathogens14060593

**Published:** 2025-06-16

**Authors:** Merve Ozturk, Muhammet Bahaeddin Dortbudak, Bayram Bekmez, Lucia Biagini, Nuri Altuğ, Giacomo Rossi, Yasin Ozturk, Alessandro Di Cerbo

**Affiliations:** 1 Department of Internal Medicine, Faculty of Veterinary Medicine, University of Necmettin Erbakan, 42310 Ereğli, Konya, Turkey; merve.ozturk@erbakan.edu.tr (M.O.); nurialtug@gmail.com (N.A.); 2 Department of Pathology, Faculty of Veterinary Medicine, Harran University, 63510 Şanlıurfa, Şanlıurfa, Turkey; mbdortbudak@harran.edu.tr (M.B.D.); bekmezbayram107@gmail.com (B.B.); 3 School of Biosciences and Veterinary Medicine, University of Camerino, 62024 Matelica, Italy; giacomo.rossi@unicam.it (G.R.); alessandro.dicerbo@unicam.it (A.D.C.); 4 Department of Pharmacology and Toxicology, Faculty of Veterinary Medicine, University of Necmettin Erbakan, 42310 Ereğli, Konya, Turkey; yasinozturk@erbakan.edu.tr

**Keywords:** *Mycobacterium avium* subspecies *paratuberculosis*, Johne’s disease, oxidative stress, antioxidants, apoptosis

## Abstract

Paratuberculosis, caused by *Mycobacterium avium* subspecies *paratuberculosis* (MAP), is a chronic granulomatous enteritis with significant implications for ruminant health, economic productivity, and potential zoonotic risk. This study investigated the expression of biomarkers of oxidative stress and apoptosis in goats naturally infected with MAP, focusing on three biological matrices: serum, intestinal mucosa, and mesenteric lymph nodes. Twenty MAP-positive goats and ten healthy controls were included. Serum and tissue levels of malondialdehyde (MDA), glutathione S-transferase (GST), glutathione peroxidase (GPX), superoxide dismutase (SOD), glutathione reductase (GSR), and caspase-3 were quantitatively assessed using ELISA tests. Gross and histopathological analyses confirmed MAP infection. Infected animals showed significantly elevated serum levels of MDA and caspase-3 (*p* < 0.001), along with decreased antioxidant enzyme activities (GSR, GST, GPX, SOD). Tissue analysis revealed increased MDA and caspase-3 levels, particularly in the intestinal mucosa compared to mesenteric lymph nodes, suggesting localized oxidative damage and apoptosis. Conversely, antioxidant enzyme activity was higher in mesenteric lymph nodes, indicating a compensatory response and a pronounced involvement of the intestinal tract. These findings demonstrate that MAP infection induces marked oxidative stress and apoptotic processes, especially in the intestinal mucosa. The imbalance between pro-oxidant and antioxidant systems may play a key role in the pathogenesis and chronic progression of the disease. Caspase-3 and MDA, in particular, have been identified as promising diagnostic or prognostic biomarkers for MAP infection. This study highlights the importance of developing improved diagnostic tools and therapeutic strategies targeting oxidative stress pathways in paratuberculosis.

## 1. Introduction

Paratuberculosis, also known as Johne’s disease, is a chronic granulomatous enteric infection that predominantly affects domestic and wild ruminants. The etiological agent of the disease is the pathogen *Mycobacterium avium* subspecies *paratuberculosis* (MAP), which belongs to the *M. avium* complex (MAC). This disease, which causes chronic, progressive, and granulomatous enteritis in ruminants, has prompted considerable concern within the global livestock-rearing industry, given its substantial economic impact and its capacity for persistence within herds [1]. In addition to its potential implications for animal health, MAP poses a substantial public health risk. In human beings, MAP infection has been associated with autoimmune diseases such as Crohn’s disease, multiple sclerosis, and rheumatoid arthritis, raising concern among researchers, especially considering the risk of developing occupational diseases [2,3].

The prevalence of subclinical infection is widespread in domestic livestock, particularly cattle, sheep, and goats. MAP is spread both by animals with clinical signs and by those with subclinical infection, increasing the probability of experiencing clinical mastitis [4,5]. The main obstacles in diagnosing MAP are reported to be the non-specificity of symptoms, the chronic nature of the disease, and the delay in development of sero-immunity [6]. As elevated bacterial excretion of MAP in milk and its resistance to several of the processes employed in the dairy industry have been demonstrated [7,8,9], it is essential to implement early diagnosis to control the disease and prevent its spread [7,8,10].

The management of the disease poses a considerable challenge, primarily due to the fact that vaccines do not always guarantee immunity, as is the case with available serological tests. Moreover, management measures on the farm are difficult, and endemic infections continue to infect new herds [11,12].

In animals exhibiting clinical signs of infection, the MAP load attains elevated concentrations, thereby enabling a diagnosis by analyzing fecal cultures, milk, or colostrum by polymerase chain reaction (PCR) or ELISA test for antibody detection [13].

Paratuberculosis is characterized by a typical diffuse granulomatous infiltration of the intestinal lamina propria, largely restricted to the ileum and particularly to the ileocecal valve region of the small intestine [14]. The main histopathological lesions reported include granulomatous enteritis, granulomatous lymphadenitis of mesenteric and ileocecal lymph nodes, and lymphangitis [15]. The clinical signs associated with MAP infection are largely due to chronic inflammation. A salient feature that distinguishes MAP from other mycobacterial infections is the distribution of inflammation as diffuse. In contrast, the latter are instead characterized by a typical nodular, tuberculoid, granulomatous inflammation, as observed in the case of *Mycobacterium bovis* or *Mycobacterium tuberculosis*. The formation of granulomas in response to mycobacterial infection is reported as a physiological response to limit the dissemination of the mycobacterium. However, it has been demonstrated that the trafficking of macrophages inside infected granulomas and the phagocytosis of mycobacteria are directly involved in the spread of the disease and the formation of secondary granulomas [16]. In the case of *M. avium* subsp. *paratuberculosis*, following an initial T-cell response, characterized by proinflammatory cytokines such as interferon gamma (IFN-γ), interleukin-1α (IL-1α), IL-6, and IL-2, which would favor the formation of granulomas, there is a shift to a typical humoral Th2 response characterized by enhanced expression of IL-4, IL-5, and IL-10 [17]. This immune response has been demonstrated to be ineffective in controlling the spread and replication of the mycobacterium. Instead, it has been shown to increase the infiltration of macrophages and inflammation, thus shifting the condition to a chronic state [18]. A salient factor contributing to the elusiveness of MAP is its capacity to persist within the confines of host macrophages. Macrophages are professional phagocytes responsible for the removal and destruction of foreign bodies within the host organism. MAP impedes the maturation of normal phagosomes, thus enabling bacterial survival within stalled phagosomes, which act as reservoirs for the subsequent proliferation of bacteria [19]. The process of apoptosis of infected macrophages could be considered a protective mechanism against mycobacterial infection.

Nevertheless, it has been demonstrated that mycobacteria can evade this response by inhibiting apoptosis and stimulating necrosis, thereby facilitating the further dissemination of the pathogen [20]. Furthermore, the alteration of the apoptotic pathway during an active infection, in conjunction with the diminished capacity to eliminate apoptotic cells, can result in the apoptotic cell bodies losing their membrane integrity and becoming secondary necrotic cells [21]. When this mechanism involves an active macrophage, the consequence includes the leakage of lysosomal content and reactive oxygen species (ROS), leading to systemic inflammation and oxidative stress, thereby stimulating surrounding cell apoptosis and inflammation [22].

Intense oxidation, tissue damage, and apoptosis have been reported in animals affected by paratuberculosis [23,24]. Indeed, MAP infection can induce a broad spectrum of chemical reactions and metabolic pathways concomitant with inflammation [25]. Reactive oxygen and nitrogen species encompass a range of molecules, including highly reactive molecules such as the superoxide anion (O_2_-), the hydroxyl radical (OH-), and the peroxynitrite (ONOO-), as well as more stable oxidants, such as hydrogen peroxide (H_2_O_2_) and nitric oxide (NO). These products are the result of autooxidative metabolic processes. The excessive accumulation of these radicals has been demonstrated to lead to oxidative stress, the release of inflammatory mediators, and their interaction with endothelial cells, thereby initiating the apoptosis pathway [26]. Malondialdehyde (MDA) is the principal and most studied polyunsaturated fatty acid peroxidation product. This exhibits toxic effects by binding to nucleic acids, phospholipids, and amino groups of proteins, showing mutagenic and carcinogenic features [27]. The response to oxidative stress is considered an important biomarker of the protective ability against infection-induced damage and the activation of the immune system [28]. Superoxide dismutase (SOD) and glutathione peroxidase (GPx) represent some of the most significant antioxidant enzymes, whose function is to impede the accumulation of free radicals that are released during periods of oxidative stress and the onset of lipid peroxidation. SOD catalyzes the conversion of superoxide to hydrogen peroxide, while GPx removes hydrogen peroxide produced by SOD from tissues. It has been demonstrated that a deficiency or reduction in the availability of antioxidants is concomitant with an accumulation of free radicals. This, in turn, has the potential to compromise and disrupt the structural stability and functional capabilities of cell membranes [23].

Given the considerable economic losses attributable to MAP infection and its potential threat to human health, there is an urgent need to develop rapid and effective methods to detect the infection. It was hypothesized that the chronic inflammatory process induced by MAP infection alters the functionality of antioxidant enzymes due to increased production of free radicals, thereby contributing to oxidative stress and influencing apoptosis rates. Therefore, the objective of the present study was to determine the serum and tissue concentrations of malondialdehyde, superoxide dismutase, glutathione peroxidase, glutathione S-transferase, glutathione reductase, and caspase-3 in MAP-infected animals.

## 2. Materials and Methods

### 2.1. Ethical Statements and Study Design

The present study comprised 20 hair goats that exhibited severe emaciation and chronic diarrhea. These animals had not been vaccinated against paratuberculosis and were found to be positive for *Mycobacterium avium* subsp. *paratuberculosis* (MAP). A control group of 10 healthy hair goats that were negative for MAP was also included in the study. Animals were screened for MAP infection using commercial ELISA tests (See Section 2.2). The number of animals included in this study (20 MAP-infected and 10 healthy controls) was determined based on ethical considerations and statistical requirements. The study design adhered to the reduction principle by minimizing the number of animals used while ensuring the reliability and validity of the findings. The study was conducted with the approval of Bingöl University Animal Experiments Local Ethics Committee (BÜHADYEK 02/13 dated 31 March 2023).

### 2.2. ELISA Screening Test for Antibodies to Mycobacterium avium ssp. paratuberculosis

In order to detect the positivity for MAP infections, serum samples were analyzed using a specific commercial ELISA kit for goat paratuberculosis (Paracheck 2, No. 63325, Prionics AG, Zurich, Switzerland) to detect antibodies to *Mycobacterium avium* subsp. *paratuberculosis*, according to the recommended procedure (sensitivity 65–88%). The absorbance/optical density was measured using a spectrophotometric plate reader, employing a 450 nm filter (Rayto RT-2100C, Shenzhen, China). The diagnosis was further confirmed with histopathological examination (See Section 2.4 and Section 3.1).

### 2.3. Blood Serum Collection, Oxidative Stress, and Apoptosis Biomarkers

An 8 mL blood sample was collected from the vena jugularis of all goats included in the study using anticoagulated tubes (BD Vacutainer^®^, Franklin Lakes, NJ, USA) before slaughtering. The samples were allowed to clot for approximately one hour at room temperature. The serum samples were centrifuged at 3000 rpm for 10 min (Hermle Z 36 HK^®^, Wehingen, Germany). The sera obtained were stored at −20 °C until analysis. In the present study, the ELISA kits indicated in Table 1 were both oxidative stress biomarkers (MDA, GSR, GST, GPX1, SOD). In addition, the caspase-3 kit was employed as a marker of apoptosis. All the kits were used according to the Mybiosource ELISA test kit protocols.

### 2.4. Biological Sample Collection and Histopathological Analysis

The MAP-infected animals were slaughtered to prevent environmental contamination. All animals were subjected to necropsies, and tissues were collected for further analysis. Furthermore, the animals in the control group were slaughtered at the end of the production cycle. Only the organs were taken for further analysis.

Samples of intestinal and lymph node tissue were subjected to homogenization, after which the resultant homogenates were stored at a temperature of −20 °C until required. The tissue levels of caspase-3 were determined as an apoptosis biomarker, and MDA, SOD, GSR, GST, and GPX were determined as oxidative stress markers, as previously described for serum samples. Furthermore, a proportion of the tissue samples were fixed in 10% buffered formalin and then routinely processed for histopathological examinations [29]. The samples were dehydrated through a graded series of alcohol and cleared with ethanol/xylene solution before being embedded in paraffin wax. Serial sections (5 μm thick) were cut with a rotary microtome (Leica RM2135, Wetzlar, Germany) on normal and polylysine slides. The slides were then stained with hematoxylin–eosin (HE) and Ziehl–Neelsen (ZN) staining and mounted for observation under the optical microscope (Leica DM2500, Wetzlar, Germany).

### 2.5. Statistical Analysis

Data were analyzed using GraphPad Prism 9.5.1 software (GraphPad Software Inc., La Jolla, CA, USA). All data are presented as means ± standard deviation. A Shapiro–Wilk test was applied to analyze sample normality. Differences in serum oxidative stress and apoptosis biomarkers in healthy and infected goats and intestinal mucosa and mesenteric lymph nodes homogenates were analyzed using an unpaired *t*-test. Moreover, a Pearson correlation was applied to serum, intestinal mucosa, and mesenteric lymph nodes stress and apoptosis biomarkers of infected goats. A *p* < 0.05 was considered significant.

## 3. Results

### 3.1. Macroscopic and Histopathological Findings

**Figure 1:**
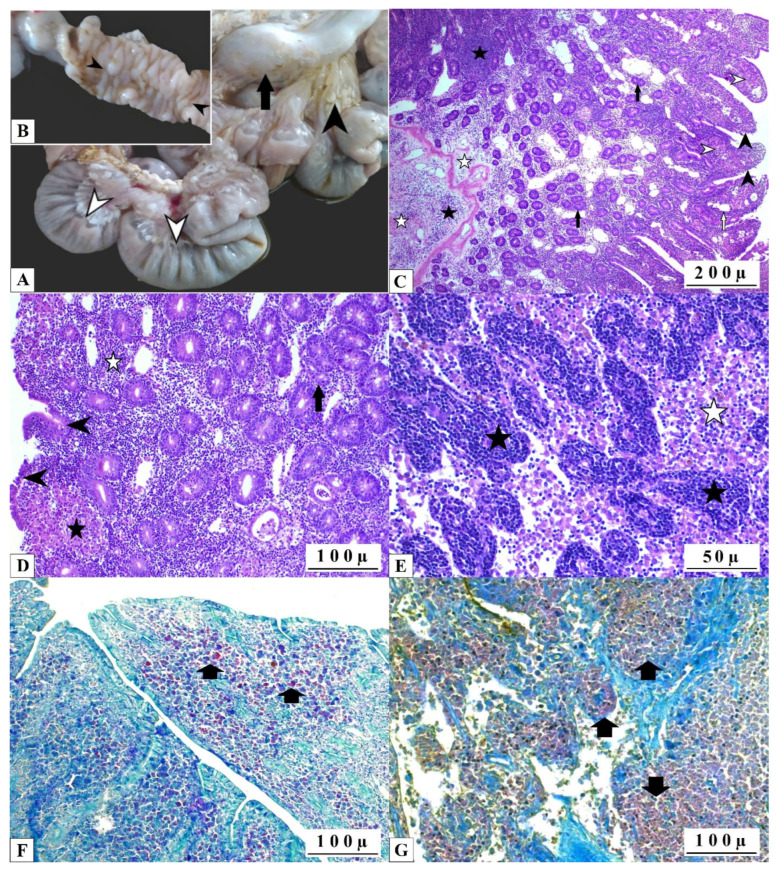
Gross and histological aspect of MAP-positive goats’ intestines. (**A**) Intestinal tortuosity (white arrowheads), serous atrophy of the mesenterium (arrowhead), excessive growth of the mesenteric lymph node (arrow). (**B**) Thickening and tortuosity of the intestinal mucosa (arrowheads). (**C**) Atrophy and degeneration of intestinal villi (arrowheads), diffuse infiltration of epithelioid histiocytes (white arrowheads), dilatation of villous lacteals (withe arrow), proliferation of mucosal crypt glands (arrows), mononuclear leukocyte infiltrations (stars) associated with edema and fibrosis (hollow stars) in the mucosa and submucosa, scale bar 200 microns, H&E. (**D**) Degenerative and necrotic changes in villous epithelial cells (arrowheads), proliferation of mucosal crypt glands (arrows), infiltration of epithelioid macrophages in the lamina propria (stars) and mononuclear leukocyte infiltrations (white stars), scale bar 100 microns, H&E. (**E**) Epithelioid macrophages (white star) and lymphoproliferative foci (star) in mesenteric lymph nodes, scale bar 50 microns, H&E. (**F**) Acid-fast agents in lamina propria (arrows), scale bar 100 microns, ZN. (**G**) Acid-fast agents in mediastinal lymph nodes (arrows), scale bar 100 microns, ZN.

Macroscopic examination of the organs obtained from infected goats sent for slaughtering revealed characteristic lesions of paratuberculosis in the intestines and associated lymph nodes. The mean volume of serous or serofibrinous exudate observed within the abdominal cavities was 2–3 L. Mesenterium, omentum, and subperitoneal adipose tissue were found to be atrophied and replaced by diffuse yellowish gelatinous edema. The intestinal serosa exhibited a diffuse edema and thickening of the mucosal layer, resulting in folds that resisted straightening when subjected to traction. Although the majority of intestinal segments were affected, the lesions were predominantly located in the ileum, jejunum, and, to a lesser extent, in the proximal section of the caecum. The lymph vessels exhibited thickening, forming cords, and the mesenteric lymph nodes demonstrated enlargement. Transverse sections of edematous lymph nodes revealed that the cortex–medulla distinction was indistinguishable (Figure 1A,B).

Histopathological examination of the intestinal section revealed a diffuse granulomatous inflammation, which was associated with villus atrophy, fusion, and degenerative-necrotic modification of the villus surface epithelium. The entire layer of intestinal mucosa was affected by edema and severe leukocyte infiltration, which was dominated by epithelioid macrophages. As demonstrated in Figure 1C,D, the submucosal layer exhibited signs of severe edema and fibrosis, in addition to mild leukocyte infiltration. In the mesenteric lymph nodes, dilatation of the paracortical sinuses, lymphoproliferation of the subcapsular follicles, and epithelioid macrophages were observed (Figure 1E). The Ziehl–Neelsen (ZN) staining revealed the presence of numerous bright red, acid-fast mycobacteria in the intestinal lamina propria, epithelioid histiocytes, and extracellular mesenteric lymph nodes (Figure 1F,G).

### 3.2. Blood Serum Oxidative Stress and Apoptosis Biomarker

**Figure 2:**
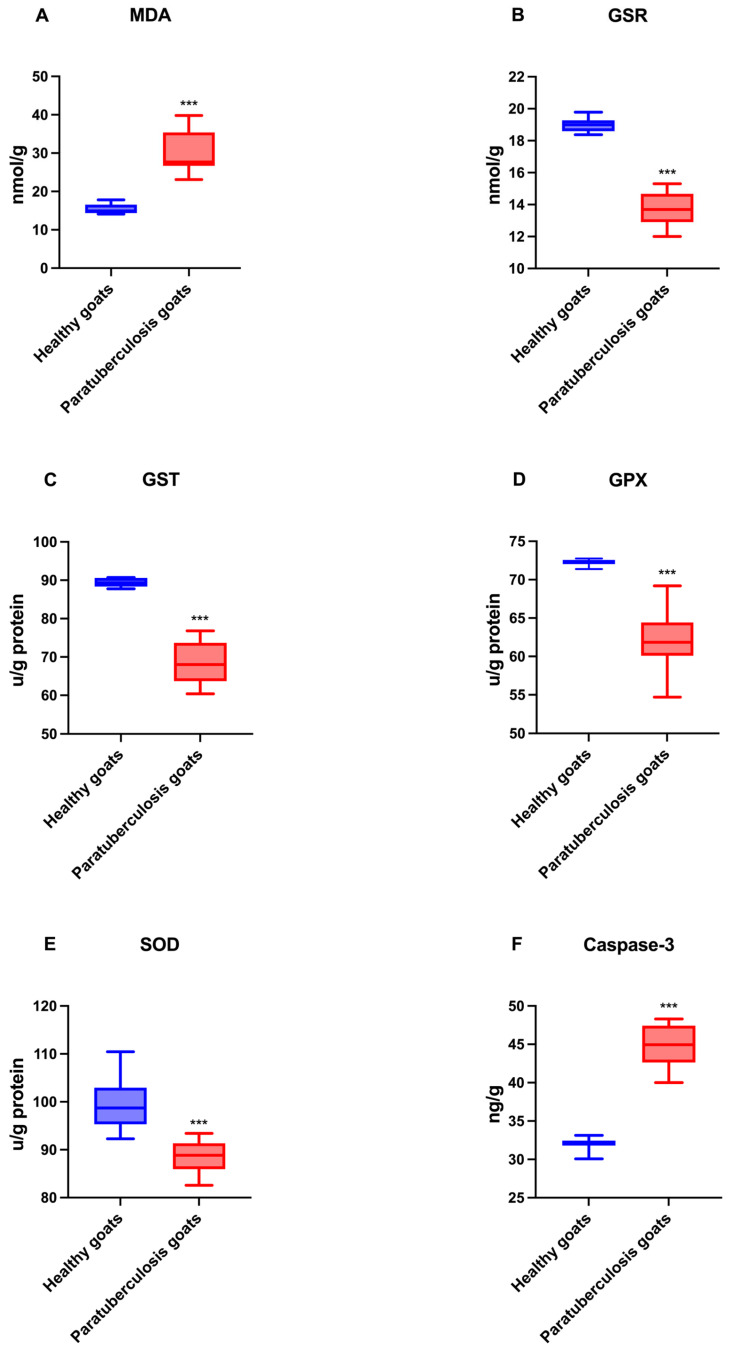
Graphical representation of oxidative stress (**A**–**E**) and apoptosis biomarker (**F**) levels in sera of healthy and infected goats. *** *p* < 0.001.

The values for oxidative stress and apoptosis biomarkers are presented in Figure 2. Serum concentrations of MDA and caspase-3 were significantly higher in goats with paratuberculosis compared to healthy goats (*p* < 0.001). Conversely, serum concentrations of GSR, GST, GPX, and SOD were found to be significantly lower in goats with paratuberculosis compared to healthy goats (*p* < 0.001).

### 3.3. Oxidative Stress and Apoptosis Biomarkers in Intestinal Mucosa and Mesenteric Lymph Nodes

**Figure 3:**
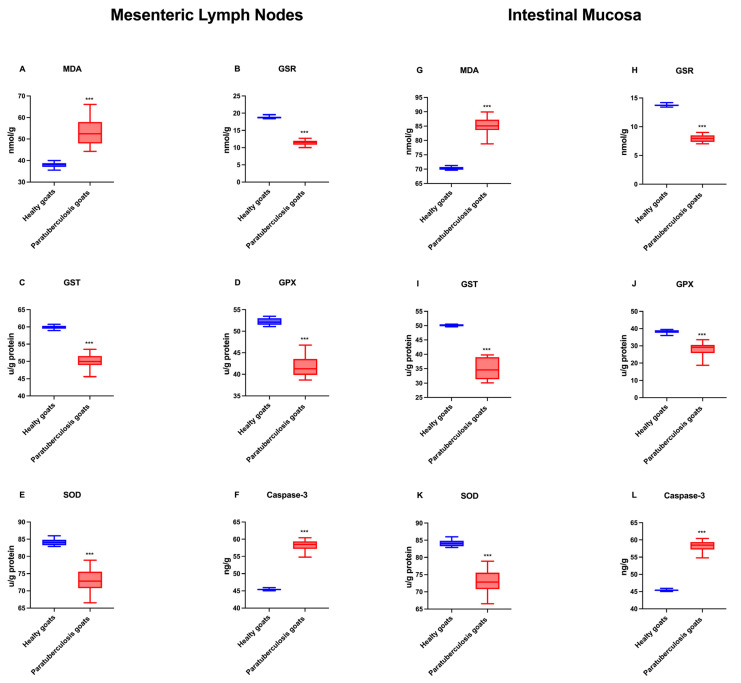
Graphical representation of oxidative stress and apoptosis biomarker levels in (**A**–**F**) mesenteric lymph nodes and (**G**–**L**) intestinal mucosa homogenates in healthy and infected goats. *** *p* < 0.001.

The mean values of oxidative stress and apoptosis biomarkers in the mesenteric lymph nodes and intestinal mucosa of MAP-infected goats and control goats are shown in Figure 3. Focusing on MAP-infected goats, intestinal samples exhibited elevated levels of MDA and caspase-3 compared to mesenteric lymph nodes, while GSR, GST, GPX, and SOD values showed higher values in mesenteric lymph node compared to intestinal mucosa (*p* < 0.001). Both the intestinal mucosa and the lymph nodes demonstrated increased levels of MDA and caspase-3 compared to the control group (*p* < 0.001). Conversely, GSR, GST, GPX, and SOD levels were more expressed within the control group (*p* < 0.001).

### 3.4. Correlation Among Serum, Intestinal Mucosa, and Mesenteric Lymph Nodes Stress and Apoptosis Biomarkers of Infected Goats

**Figure 4:**
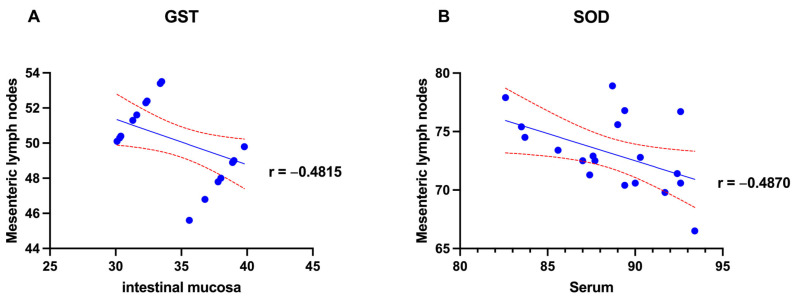
Graphical representation of correlation between (**A**) intestinal mucosa and mesenteric lymph nodes of GST values (*p* < 0.05), and (**B**) serum and mesenteric lymph nodes SOD values. *p* < 0.05.

Among all the parameters analyzed in this study, significant correlations were observed only for GST and SOD values. Specifically, Pearson correlation analysis revealed a significant negative correlation between intestinal mucosa and mesenteric lymph node GST levels (r = −0.4815, *p* < 0.05), as well as between serum and mesenteric lymph node SOD levels (r = −0.4870, *p* < 0.05) (Figure 4).

## 4. Discussion

Bacteria belonging to the *Mycobacterium* genus are classified as Gram-positive, acid-fast organisms, including several significant human and animal pathogens. *M. avium* subsp. *paratuberculosis* is the etiological agent of Johne’s disease, a severe chronic gastroenteritis affecting ruminants. Infection by MAP induces severe chronic inflammation in tissues, primarily involving the gastrointestinal tract and mesenteric lymph nodes. The ability of the microorganism to spread and survive within the host is contingent on its capacity to circumvent the activity of macrophages, which are the target of the infection. This is achieved by avoiding antigen processing by inhibiting phagolysosomal fusion and acidification [30]. Mycobacteria demonstrate high resistance to bactericidal mechanisms, such as reactive oxygen species, through catalase and peroxidase activities [30]. The ineffectiveness of the granulomatous inflammatory response is the underlying cause of progressive tissue damage and chronicization of the condition. Phagocytic activation has been demonstrated to generate free radicals, with these reactive oxygen species contributing to the exacerbation of tissue damage [31,32]. Such damage can lead to several subsequent effects, including lipid peroxidation, disruption of membrane integrity, and inactivation of membrane proteins, receptors, and enzymes that bind to them.

The elevated serum MDA levels in MAP-infected goats is an indication of enhanced cell damage and lipid peroxidation. Malondialdehyde is the most reactive dialdehyde, derived from different processes such as the peroxidation of polyunsaturated fatty acids or the metabolism of arachidonic acid [33]. The substance’s high reactivity allows it to readily combine with various functional groups found in molecules, including proteins, lipoproteins, and DNA, resulting in different chemical modifications with proinflammatory effects and mutagenic activity [27,34]. In the present study, elevated levels of malondialdehyde (MDA) were detected in the blood serum, mesenteric lymph nodes, and intestinal mucosa of naturally MAP-infected goats compared to healthy animals. This finding suggests that oxidative damage occurred due to an increase in free radicals. Furthermore, the higher level of MDA in the intestinal mucosa compared to the mesenteric lymph nodes indicates that the intestinal mucosa of infected goats was more severely affected by the infection than mesenteric lymph nodes.

Antioxidants are enzymes involved in controlling reactive oxygen species (ROS) and reactive nitrogen species (RNS) levels, which counteract their downstream cellular effects of excessive oxidation [35]. Under physiological conditions, pro-oxidant over antioxidant compounds have a moderate preponderance. This has been shown to result in a mild form of oxidative stress, the resolution of which is contingent on the body’s inherent antioxidant systems [36]. Intracellular antioxidants include enzymes such as SOD, GSR, and GST [35]. It was hypothesized that the chronic inflammatory process induced by MAP infection may alter the functionality of antioxidant enzymes due to the increase in free radicals generated. Serum and tissue SOD concentration in goats with paratuberculosis was found to be lower than in the control group. Similarly, previous studies indicated that low serum SOD levels may be attributable to antioxidant suppression arising from oxidation [37,38]. Tissue SOD levels were found to be lower in the intestinal mucosa than in the mesenteric lymph node, supporting the idea that more severe tissue damage occurs in the intestinal mucosa.

Reduced glutathione (GSH) is an essential non-enzymatic antioxidant in mammalian cells. It has been demonstrated that GSH fulfils a dual role, functioning as both an antioxidant and a cofactor for various enzymes implicated in antioxidant and detoxification processes. These include glutathione peroxidases, glutathione S-transferases, and glyoxalases [39]. GSH depletion and post-translational modifications of proteins through glutathionylation activity are critical regulators of apoptosis; therefore, multiple metabolic problems can derive from GSH depletion [40]. Furthermore, GSH has been employed in clinical settings to diagnose hepatic and oncological diseases, assess riboflavin deficiency, and identify specific genetic disorders [41]. The present study set out to determine whether there was a discrepancy in the GSH levels of MAP-positive goats compared to healthy goats. The results obtained demonstrated that the levels of GSH in the blood serum and tissues of MAP-positive goats were lower than those in the serum of healthy goats. However, the tissue expression of GSH in the mediastinal lymph nodes was higher than in the intestinal tissue. This finding suggests that the damage to the intestinal tissue is more severe.

Glutathione S-transferases (GST) are a multifunctional enzyme family that plays a pivotal role in detoxifying reactive metabolites by catalyzing their conjugation with reduced glutathione. They play a role in the intracellular transport of compounds and their subsequent delivery to sites for transformation and/or excretion [42]. In the present study, it was observed that the serum GST levels of healthy goats were found to be higher than those of MAP-positive goats, and higher tissue levels were detected in the mediastinal lymph node than in the intestinal tissue of infected animals.

Glutathione peroxidases (GPxs) belong to a family of phylogenetically related enzymes known to catalyze the reduction of H_2_O_2_ or organic hydroperoxides to water or the corresponding alcohols, respectively, typically using glutathione (GSH) as a reductant [43]. This process is known to function with superoxide dismutase and catalase, ensuring the maintenance of the redox balance within mammalian cells [44]. It was observed that serum GPX levels detected in MAP-infected goats were lower than in healthy goats and in the intestinal mucosa compared to the lymph nodes. A comparison of serum and tissue GPX levels revealed that the serum exhibited a higher concentration.

The decline in the levels of SOD, GPx, and GST in both serum and organs of infected animals indicates the role of MAP infection in causing significant disruption to the redox balance. In addition, the antioxidant defense system is inadequate due to increased oxidative stress. This finding lends further support to the results of the study conducted by Balikci and Gurdogan (2015), which stated that oxidative stress parameters such as SOD, GPx, and GSH increase during paratuberculosis infection due to their role in the transformation of radicals into less effective metabolites [37].

Moreover, the significant inverse correlations observed between antioxidant enzyme levels in different tissues—specifically between GST in the intestinal mucosa and mesenteric lymph nodes, and between SOD in serum and mesenteric lymph nodes—may reflect a tissue-specific redistribution of oxidative stress responses in animals affected by MAP. As observed, MAP infection induces a chronic granulomatous enteritis characterized by persistent inflammation, macrophage activation, and local immune cell infiltration, particularly in the intestinal tract and associated lymphoid tissues. These inflammatory processes are known to generate high levels of ROS, necessitating the upregulation of endogenous antioxidant defenses. The negative correlations described may indicate a shift from systemic to local antioxidant defense, as the immune response becomes increasingly compartmentalized in chronically inflamed lymphoid tissues [45]. This redistribution of antioxidant enzymes may represent a pathological adaptation aimed at preserving immune cell function in lymph nodes under sustained oxidative stress, while inadvertently compromising antioxidant capacity in peripheral compartments. Such tissue-specific imbalances could contribute to the progression of mucosal damage, increased intestinal permeability, and systemic oxidative burden frequently observed in advanced stages of paratuberculosis [46].

Detecting active caspases within a tissue is a valuable method for evaluating both early and late stages of apoptosis. Caspases are aspartate-specific cysteine proteases, which cleave their substrates on the carboxyl side of the aspartate residue. These are divided into two main groups, the initiator caspases (e.g., caspases 8, 9, and 10) and the effector caspases (e.g., caspases 3, 6, and 7). Caspase-3 is the most studied effector caspase and is involved in extrinsic and mitochondrial apoptosis pathways [47]. Serum caspase-3 levels have been regarded as a negative prognostic biomarker in the course of a disease [48]. The present study has demonstrated that goats affected by paratuberculosis exhibit higher levels of caspase-3 in their serum samples, thereby validating the hypothesis that this condition is associated with a severe form of apoptosis.

Furthermore, a comparison of the levels of caspase-3 in the mesenteric lymph node and the small intestinal mucosa reveals that higher levels of caspase-3 expression were detected in infected goats and that intestinal mucosa was more severely damaged when compared to the mesenteric lymph node. A previous study indicated the evaluation of caspase-3 as a biomarker for active human tuberculosis [49]. In addition, De Matteis et al. (2023) reported that caspase-3 detection is an additional bovine tuberculosis biomarker for buffalos with paratuberculosis [50]. The results of this study corroborate the previous outcomes, showing an increase in caspase-3 levels in serum, intestine, and mesenteric lymph node of MAP-infected hair goats.

The macroscopic findings observed in the necropsy of animals infected with MAP allowed further confirmation of the disease, highlighting its main features in the various organs. Whilst most intestinal infections in domestic ruminants are of an erosive–ulcerative or exudative character, a proliferative diffuse granulomatous inflammation was observed in the tissues of MAP infection [51]. The macroscopic findings exhibited severe thickening of the intestinal mucosa, attributable to a dense infiltrate of granulomatous inflammatory cells in the lamina propria, as well as rough, rugose mucosa, frequently accompanied by multiple foci of ulceration, and enlarged mesenteric lymph nodes, all findings in accordance with previous studies [52,53]. Histopathological evaluation confirmed the gross examination findings, revealing a diffuse granulomatous inflammatory infiltrate expanding the intestinal lamina propria, associated with fibrosis and partially edema-related thickening area, villus loss, and atrophy, all histopathological findings typical of the condition, confirmed by Zhiel–Nielsen stain [54].

This study aimed to identify markers with the capacity to provide relevant indications of the infectious state and act as prognostic indicators for the disease. The preliminary results obtained confirm the initial hypothesis, as it was observed that infection causes an increase in oxidation products, such as malondialdehyde (MDA), and a decrease in enzymes involved in protecting against free radical damage. This process is associated with the induction of apoptosis, directly by replicating the microorganism and the chronic inflammation in the tissue.

## 5. Conclusions

The infection and multiplication of animal populations in high-risk areas for ruminant animals regarding MAP infection have not yet been fully prevented. Currently, there is no pharmaceutical agent available to treat this condition. The primary measures employed to combat the disease include vaccination of the host within the ruminant to enhance its resistance to infection, environmental hygiene management to eradicate MAP bacteria in the ruminant’s immediate environment, and the utilization of diagnostic tests and methodologies to identify and eliminate the sources of MAP infection within the herd. Although this study did not directly assess clinical outcomes or disease progression, the elevated levels of malondialdehyde and caspase-3, alongside reduced antioxidant enzyme activity in MAP-infected goats, suggest a state of heightened oxidative stress and apoptosis. These alterations reflect significant tissue damage and immune dysregulation, which are typically associated with advanced disease. Therefore, it can be hypothesized that such biomarker profiles may be associated with a more severe or chronic course of paratuberculosis, potentially serving as negative prognostic indicators. However, longitudinal studies are needed to validate the prognostic utility of these biomarkers.

## Figures and Tables

**Table 1:** Characteristics of the ELISA kits used in the study.

	*Catalog N.*	*Sensitivity*	*Detection Range*
*MDA*	MBS265688	0.5 nmol/mL	1.56–100 nmol/mL
*GSR*	MBS9310895	0.62 ng/mL	1.56–100 ng/mL
*GST*	MBS736956	0.056 ng/mL	0.16–10 ng/mL
*GPX1*	MBS735071	0.26 ng/ml	0.5–180 ng/ml
*SOD*	MBS8819950	0.066 U/mL	0.16–10 U/mL
*Caspase-3*	MBS737368	0.1 ng/mL	2.5–50 ng/mL

## Data Availability

All available data are shown in the manuscript.

## Abbreviations

The following abbreviations are used in this manuscript:

MAP	*Mycobacterium avium* subspecies *paratuberculosis*
MDA	Malondialdehyde
GST	Glutathione S-transferase
GPX	Glutathione peroxidase
SOD	Superoxide dismutase
GSR	Glutathione reductase
PCR	Polymerase chain reaction
IFN-γ	Interferon gamma
IL	Interleukin
HE	Hematoxylin–eosin
ZN	Ziehl–Neelsen
